# The Influence of Social Isolation and Medical Comorbidities on Geriatric Congestive Heart Failure Hospital Readmissions

**DOI:** 10.51894/001c.5959

**Published:** 2017-08-24

**Authors:** Daniel Keyes, Greg Sheremeta, Jerrit Yang, Naomi Davis, Shiling Zhang, Kevin Boehm

**Affiliations:** 1 St Mary Mercy Hospital Emergency Medicine and Graduate Medical Education Research, Livonia, MI; St Mary Mercy Hospital Department of Emergency Medicine, Livonia, MI; Michigan State University School of Osteopathic Medicine, East Lansing, MI; University of Michigan School of Medicine, Ann Arbor, MI; 2 Michigan State University School of Osteopathic Medicine, East Lansing, MI; College of Podiatric Medicine and Surgery, Des Moines University; 3 St Mary Mercy Hospital Emergency Medicine and Graduate Medical Education Research, Livonia, MI; 4 University of Texas Rio Grande Valley (UTRGV) School of Medicine, Department of Family and Community Medicine; 5 St Mary Mercy Hospital Emergency Medicine and Graduate Medical Education Research, Livonia, MI; St Mary Mercy Hospital Department of Emergency Medicine, Livonia, MI; Michigan State University School of Osteopathic Medicine, East Lansing, MI

**Keywords:** geriatric medicine, comorbidity, congestive heart failure, social isolation

## Abstract

**CONTEXT:**

Social isolation and comorbidities are likely to have a significant level of influence on the healthcare use patterns of geriatric patients with ongoing congestive heart failure (CHF)-related needs.

**METHODS:**

A retrospective study was conducted in a specialized emergency department (ED) with a sample of 286 geriatric CHF patients who initially received CHF-related care over a six-month period. Social isolation levels were assessed using a pre-existing four-point screening tool used in the study setting and composite comorbidity was gauged using the Charlson Comorbidity Index method. Subjects were categorized into either “less than 30-day readmission” or “greater than 30-day readmission/non-readmitted” sample subgroups. The setting was a single 304-bed community hospital with approximately 45,000 annual ED visits. The analytic sample was comprised of geriatric patients 65+ years of age with an ICD-9 code corresponding to CHF.

**RESULTS:**

There were no statistically significant differences between earlier hospital readmission versus later/non-readmitted sample patients when grouped by age, race, gender or level of measured social isolation. However, composite comorbidity scores were significantly lower for patients in the >30-day/non-readmitted subgroup compared to earlier readmission patients.

**CONCLUSIONS:**

These initial study results suggest that a larger proportion of CHF hospital readmissions may be more heavily influenced by clinical factors than social living arrangements. Future studies with larger samples and validated measures of social isolation are needed to inform the development and testing of programs for geriatric CHF patients striving to avoid unnecessary hospital readmissions and adverse health outcomes.

## INTRODUCTION

The geriatric population in the U.S. is rapidly growing.^1^ Geriatric patients who are 65 years and older tend to be particularly concentrated users of the health care system.^2^ This group of patients also have higher relative rates of hospital admissions and readmissions for congestive heart failure (CHF).^3^ In addition to the monetary burden placed on patients and hospitals, readmissions also often severely disrupt the well-being of CHF patients and families. A growing number of U.S. healthcare systems are beginning to brace for increased numbers of geriatric patients as a result of an aging “baby boomer” generation.^4^

During recent years, the Center for Medicare and Medicaid Services (CMS) has focused attention on higher-cost users of health care systems. An important example of this phenomenon concerns how admissions and readmissions for the diagnostic related group (DRG) amount of CHF is routinely associated with especially high healthcare costs. This chronic condition alone accounts for 6.5 million annual hospitalizations in geriatric patients and directly/indirectly accounts for 60-70% of their hospital admissions.^5^ Medicare now penalizes hospitals for excessive 30-day readmissions for CHF.^6^

For years, the CHF 30-day hospital readmission rate has been used as a key quality of care measure by CMS and served as a research focus.^3^ The cost of CHF readmissions has been estimated at $28 billion per year, with annual costs increasing significantly to $44 billion dollars for all-cause readmissions of CHF patients.^5^ In addition, it has been estimated that 75% of all-cause readmissions could be avoided, comprising an annual potential savings of $12 billion.^6^

In 2010, the Patient Protection and Affordable Care Act was signed into law containing measures to penalize hospitals for all-cause readmissions within 30 days of discharge.^6^ Therefore, a major goal for hospitals is to develop improved strategies to reduce readmission rates and avoid financial penalties. Due to the multiple complex influences frequently affecting many CHF outcomes, investigators need to continue to examine potential risk factors of CHF readmissions.^3^

One particular risk factor, “social isolation,” is a term typically used to refer to a physical separation from other people (e.g., living alone) and/or residing in a rural geographic area.^7^ Social isolation has also been more broadly defined as having few social ties that one can rely on for practical and emotional support.^8^ Age, gender, marital status, and socio-economic status may also play an influential role on perceived social isolation levels among the geriatric population.^9^

The increased prevalence of social isolation has already been well characterized in older adult populations.^8^ It has also been demonstrated that social isolation is more common in women than men, especially those who are widowed.^8^ Additionally, studies have shown that geriatric patients who experience social isolation are more likely to develop severe comorbidities including cognitive decline, infectious disease and cardiovascular disease.^9^ This could be a result of or a combination involving increased self-neglect or less access to health care, although the exact relationship remains to be more fully defined.^9^

Even though there is substantial evidence that social isolation is associated with a worse prognosis after acute myocardial infarction, there is still little evidence concerning the potential influence of social isolation on prognoses for CHF.^10,11^ Since a growing number of geriatric persons tend to possess more comorbidities with increased social isolation, our nation has an urgent need to better understand the health care usage patterns of socially isolated geriatric patients.^9^

New “specialized” emergency departments (ED) focusing on the needs of geriatric patients are becoming increasingly common in the US.^12^ These types of ED focus on the needs of geriatric patients with respect to symptomatic relief and systematic screening for more prevalent clinical comorbidities and social factors. When such screening assessments indicate unmet needs, ED professionals facilitate contact with social workers to connect geriatric patients with specific services.^13^ One factor that some new EDs screen geriatric patients for is “social connectedness,” asking questions to assess the support networks that individual patients currently feel they have.^13^

### Purpose of Study

The primary purpose of this study was to identify the relationship between social isolation levels and “earlier” (i.e., less than 30-day post-discharge) and “later” (i.e., 30 days or later) hospital readmission rates in a convenience sample of geriatric CHF patients. The authors utilized a series of questions that had already been included in the electronic health record during geriatric patient screening in the authors’ specialized ED.^12^ In addition, the authors examined the relative predictive significance of composite comorbidity levels using the Charlson Comorbidity Index (CCI) method.^13^

### Setting

The study was conducted at a single setting which was a Southeastern Michigan 304-bed community hospital ED with 45,000 annual visits. In 2012, setting ED patients were 69% White, 26% Black, 1% Asian, and 4% all other races. In August, 2010, a specialized geriatric ED opened at this site. During 2012, this specialized ED accommodated approximately 5,000 (19%) of all system ED patients. Two additional hospitals with ED were located within 20 miles of this study hospital. Before the study, senior ED staff members had received earlier geriatric health care training comprised of a web-based curriculum, and the entire ED facility had undergone redesign to accommodate the new geriatric ED. One section of the ED was designated as the “Senior ED” although any patients who were ≥ 65 years of age received the same screening.

## METHODS

The authors examined the CHF hospital readmission rates among sample geriatric patients with varied levels of social isolation based on a non-validated four-point scale used in the electronic health record at the authors’ specialized ED.^13^ Criteria to stratify patients into social isolation levels were determined by the following three screening questions in the nursing assessment and screening module: 1. “Currently unmarried,” 2. “Lives alone,” and 3. “Lacks caregiver.” Each answer was scored with one point.

For example, a patient who was married, lived with one or more others, and had a caregiver would receive a score of “0.” Hence, a lower score was interpreted as indicating a lower level of social isolation. The points from screening items were added, allowing classification of sample patients along a four-point integer scale. A score of 2 to 3 was used to define patients as socially isolated individuals. A score of 0 to 1 represented non-socially isolated individuals.

Before the study, the authors had hypothesized that CHF patients who were readmitted into the study hospital within 30 days would have overall lower social isolation scores than CHF patients possibly returning to the facility greater than 30 days after discharge. The secondary outcome of the study was to determine whether composite CCI scores would have a significant influence on predicting 30-day hospital readmission events in sample patients.

**Figure 1.**
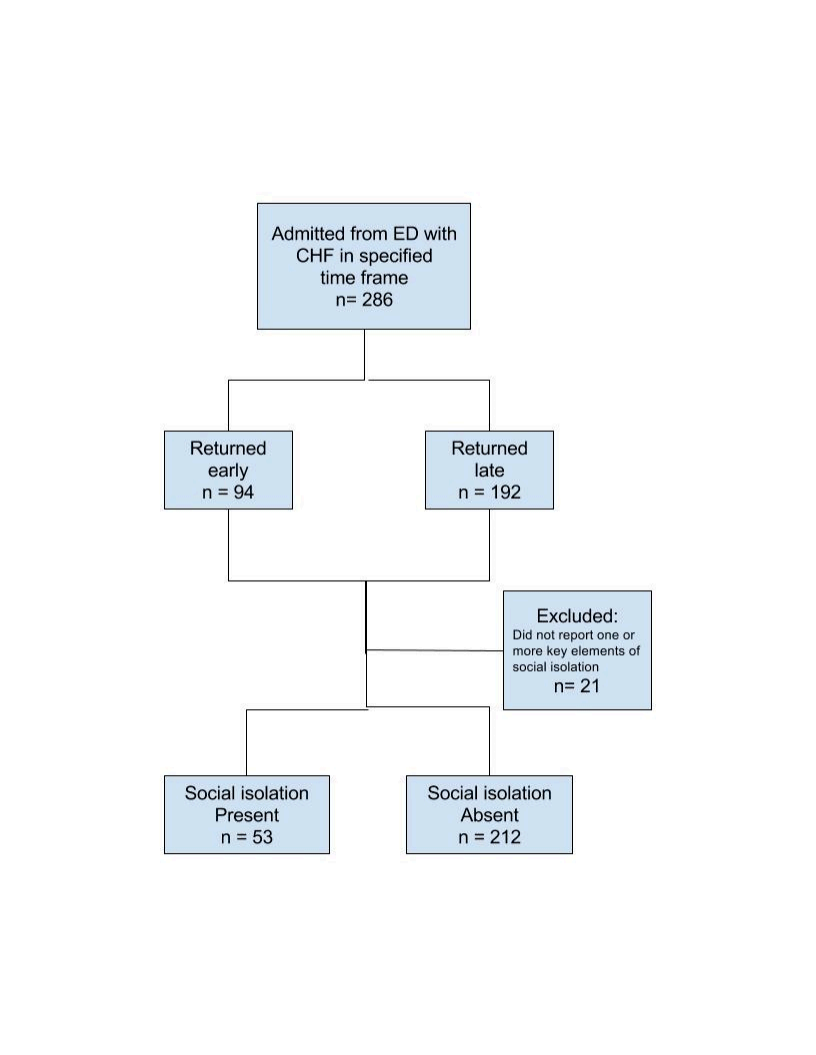
Hospital Readmission Patterns of Sample Patients

The authors conducted a retrospective chart review of geriatric patients who were first admitted to the hospital with a DRG code of CHF and stratified grouped according to their history of hospital readmission (if any). These two categories included: a) those patients who were readmitted within 30 days, and b) those who were not readmitted or readmitted after more than 30-days (Fig. 1). This stratification parameter was largely derived from the 30-day hospital readmission CMS penalties already described in this paper.

The electronic health records of geriatric CHF patients admitted during a six-month period from September 1, 2013 through February 28, 2014 were reviewed. The early hospital readmission group consisted of 94 patients readmitted with a diagnosis of CHF. The remainder of the analytic sample consisted of 192 geriatric CHF patients who were never readmitted during the six-month study window, or readmitted after greater than 30 days. The authors had already scored each patient for social isolation levels and other comorbid conditions using their data abstraction tool. Chart auditors were blinded to data concerning any possible hospital readmission events.

Patients’ composite comorbidity levels were classified using the CCI method.^13^ Although other methods exist, this method is a long-validated instrument classifying comorbidities that was first empirically developed using one-month mortality outcomes in a cohort of 604 hospital patients.^13^ The CCI classifies a patient based on their documented comorbid conditions such as diabetes, hypertension, and ischemic heart disease. Each condition is assigned a weighted score of between one through six depending on the influence of each condition on an individual’s prospective mortality risks.

Individual scores are then added together to generate a composite CCI score.^13^ By classifying comorbidities using this method, researchers are provided a validated technique of estimating the risk of mortality from comorbid diseases.^13^ Prior studies have demonstrated a correlation between higher CCI and other comorbidity score methods and increased readmission rates for heart failure.^14^

### Data Collection and Management Procedures

All study data were collected from the *Cerner®* electronic health record system of St. Mary Mercy Hospital in Livonia, MI.

The following variables were extracted from sample patients’ charts:

- Socio-demographic characteristics - age, gender and race
- Admission dates
- Confirmation of documented diagnosis of CHF
- Data concerning current living conditions (e.g., married, living alone, etc.)
- Other comorbid conditions (diseases listed in CCI method)

Items used to determine sample patients’ social isolation levels were obtained from the electronic health record “Senior Triage Risk Screening Tool” module, and marital status was obtained from the “Patient Demographics” health record screen.

The principal investigator (author DK) trained each of three chart abstractors in the proper data abstraction technique. A three-page data dictionary was created for chart abstractors’ reference and the PI remained available to address their questions. Each patient was then assigned a unique identifier accession number.

Two trained medical residents helped interpret more complex data concerning abstracted comorbid conditions. The first 20 records were abstracted with generated spreadsheets compared to identify any discrepancies for possible abstractor retraining. Double data entry was instituted throughout the study to help ensure accuracy of data collection.

### Statistical Considerations

Chart audit data were first transferred to an electronic spreadsheet and cleaned for statistical analysis. All statistical analyses were conducted using SAS, version 9.3.^15^ Inferential tests for significance of equality of two proportions of sample patient group hospital readmissions were carried out using the two tail Fisher’s exact test using GraphPad Software.^16^ Student’s t-test was used for comparing mean differences of continuous variables. A two-tailed coefficient Alpha p value of < 0.05 was observed to designate statistical significance.

The fifth author (SY) completed a series of multivariate logistic analyses to identify predictors and factors influencing readmission outcomes controlling for CCI and social isolation scores. Final stepwise methods (i.e., one model term introduced at a time) logistic regression modeling procedures were initially completed. Due to the atypical nature of this geriatric CHF patient sample, this type of “non-parametric” analytic procedure was appropriate since it was not based on any “normal” distributional assumptions. The dichotomous dependent variable was “readmission” or “later/no readmission” as defined by the 30-day cutoff.

## RESULTS

Chart data from a total of 286 geriatric CHF patients were included in the analytic sample, including 94 (32.9%) patients who were readmitted within 30 days after hospital readmission and 192 (67.1%) non-admitted or later-readmission patients (Figure 1).

Based on initial modeling results in which factors such as age, gender and race were found to be non-significant predictors, only the CCI score and social isolation score were used as independent variables in the final models. As depicted in Table 1, primary potential study influences such as age, race and gender demonstrated no statistically significant differences on readmission group differences (Table 1).

**Table 1:** **CHF Sample Patient Characteristics and Readmission Outcomes** (N = 286)

**Demographics**		**Early Readmissions** (N=94)	**Late and Non-Readmissions** (N=192)	**P-Value**
**Age**		Mean 83.3	82.4	0.4700
**CCI Score**		5.13	4.19	**0.0009**
**Gender**				0.2108
	Females	52(55.3%)	121(63.0%)	
	Males	42(44.7)	71(37.0)	
**Race**				0.4739
	White	88(93.6)	172(90.0)	
	African American	6(6.4)	19(9.9)	
	Asian	0(0.0)	1(0.52)	

P-values are the result of either two-tailed student’s t-test or chi-squared analyses.

Bold p value is statistically significant.

**Figure 2.**
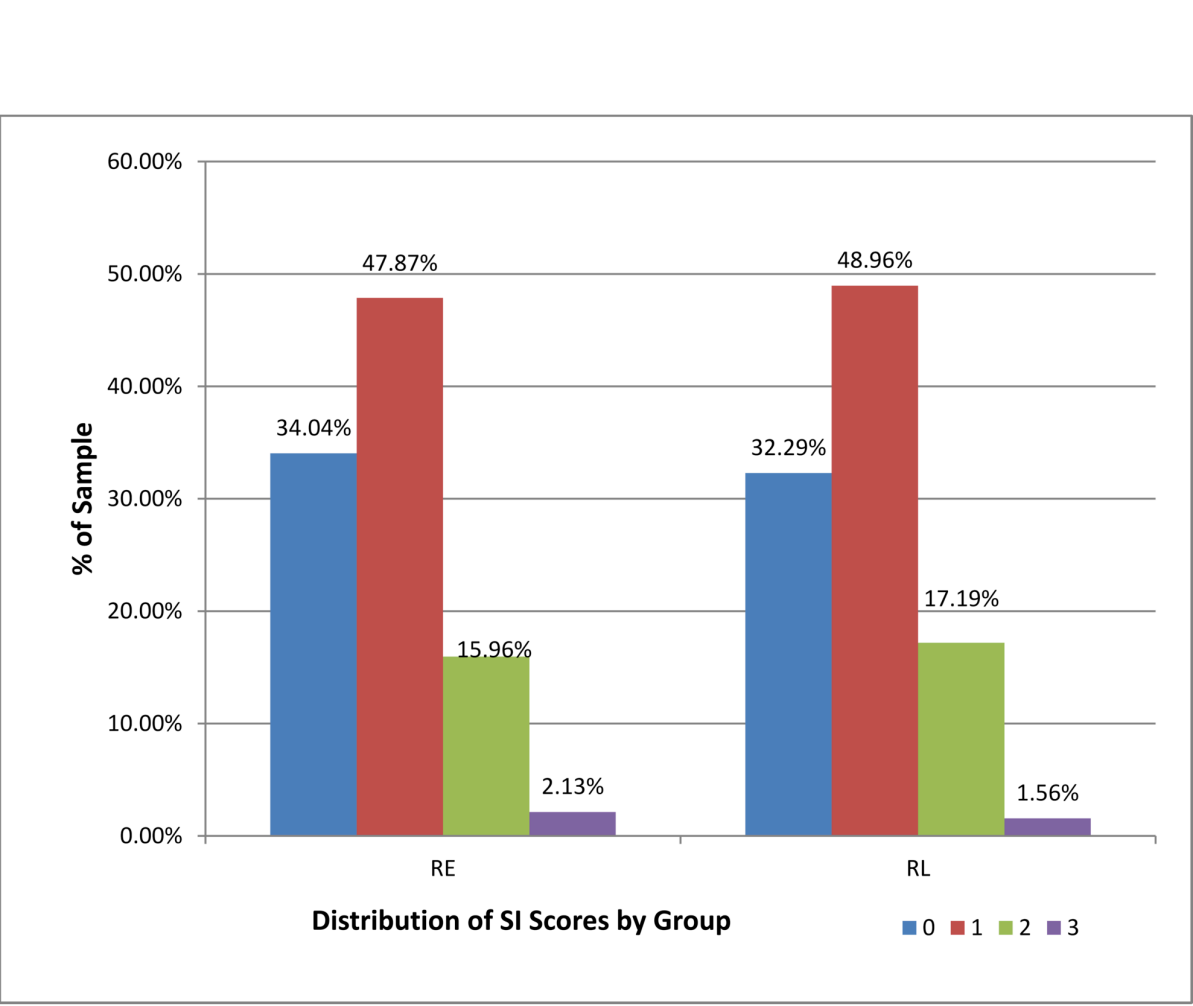
Distribution of CHF Patients by Social Isolation Scores and < 30-day Readmission (RE) and Later/non-readmitted (RL) Outcomes (N = 286)

The influence of CCI comorbidity scores, however, were significantly lower for the later readmission (i.e. listed as “RL”) subgroup (OR 0.83; 95%CI 0.75 - 0.93). However, there was no significant difference found between sub groups stratified by total isolation scores (OR 0.97; 95% CI 0.71 - 1.40) (Fig. 2 and Table 2). Neither did individual social isolation questions show significant differences: 1. No caregiver (OR 0.73; 95% CI 0.18 - 3.06), 2. Patient lives alone (OR 0.86; 95% CI 0.45 - 1.64), and 3. No current partner (OR 1.17; 95% CI 0.65 - 1.87) (Table 3).

**Table 2:** **Distribution of Social Isolation Characteristics and Readmission Outcomes** (N = 286)

		**Early Readmissions** (N=94)	**Late and Non-Readmissions** (N=192)	**P-Value**
**Lacks Caregiver**				0.9136
	Yes	18(20.9)	6(3.4)	
	No	68(79.1)	173(97.1)	
**Lives Alone**				0.7930
	Yes	18(21.0)	35(19.6)	
	No	68(79.1)	144(80.4)	
**Marital Status**				0.8133
	Married	34(36.2)	64(33.3)	
	Single/Widowed/Divorced	60(63.8)	128(66.7)	

P-values are the result of two-tailed Student’s t tests.

**Table 3:** Social Isolation Logistic Regression Analyses of Readmissions controlling for Comorbidity Scores

	**Number of patients used in analyses**	**Odds Ratio**	**95% Confidence Interval**
**Patient Lacks Caregiver**	262	0.73	0.18-3.06
**Patient Lives Alone**	265	0.86	0.45-1.64
**No Current Partner**	286	1.107	0.65-1.87
**Social Isolation Score of Earlier Readmission vs. Later/non-readmission Patients**	286	0.996	0.71-1.40

## DISCUSSION

This study examined the potential impact of social isolation on hospital readmissions in geriatric CHF patients initially screened in a specialized ED. The social isolation measure used during the study had been developed from available pre-existing screening questions used in this ED at the time of the study. No significant associations were found between social isolation scores and CHF ≤ 30-day hospital subgroup readmissions (primary outcome). Individual selected socio-demographic study measures also failed to demonstrate statistical significance.

### Study Limitations

The authors’ specific measure to evaluate social isolation levels in sample patients had not been previously validated. The use of externally validated social isolation screening tools may have required a more complex project design.

Other validated social isolation research tools certainly do exist.^14,15^ To that end, the authors are currently designing a prospective study to obtain more comprehensive screening profiles using a validated screening tool. Another challenge encountered during this current study was the limited sample size. This factor may not have provided the authors with an adequate level of statistical power to detect significant sample subgroup differences in hypothesized relationships.

It is also possible that patients who had been initially treated at the authors’ hospital were subsequently seen and readmitted at another hospital, preventing them from being counted as readmissions. However, it is quite unlikely that sample patients would have been admitted to another hospital facility given that their provider, prior testing results, and medical records were at this hospital. However, there is still no precise way of gauging what the actual magnitude of this potential issue could have been.

One key characteristic of a specialized senior ED is the systematic use of screening tools.^12,17^ Such screening includes a variety of tests for common comorbid conditions of geriatric patients. In this study, we did demonstrate that the CCI comorbidity scores were significant predictors of readmissions for sample patients, although this was not the primary outcome of the study. Other studies have demonstrated benefit from implementation of a “case management” approach to higher readmission populations during which individuals are identified and specific interventions applied.^16,17^ These geriatric patients typically receive systematic reviews of their medication use, and earlier follow up with their respective primary care physician or other subspecialist.^17,18^ Providers who implement such interventions may help to prevent more frequent ED visits and early hospital readmissions for CHF patients by improving their overall quality of life.^17^

## CONCLUSIONS

Based on these initial study results, the influence of social isolation levels on hospital readmission rates among geriatric CHF patients may be less than that from overall comorbidity.  Patients with higher CCI comorbidity levels were, significantly more likely to be readmitted to the hospital within 30 days. Future studies with larger samples and validated social isolation measures are needed to inform the development and testing of programs assisting geriatric CHF patients avoid unnecessary hospital readmissions and related adverse health outcomes.

### Conflict of Interest

The authors declare no conflict of interest.
